# Medication non-adherence in re-admitted patients at a psychiatry hospital: A qualitative study

**DOI:** 10.4102/sajpsychiatry.v31i0.2345

**Published:** 2025-02-12

**Authors:** Gopolang E. Zwide, Zukiswa Tsolekile Dewet, Funeka B. Sokudela

**Affiliations:** 1 Department of Psychiatry, Faculty of Health Sciences, University of Pretoria, Pretoria, South Africa

**Keywords:** medication non-adherence, psychiatric patients, readmission, relapse prevention, substance abuse, health literacy

## Abstract

**Background:**

Medication non-adherence is a significant public health concern and is prevalent among mental healthcare users. Approximately 65% of patients with severe mental illness do not adhere to their prescribed medication. Medication nonadherence may worsen mental illness and result in poorer clinical outcomes, including frequent relapses and rehospitalisation rates, as well as long time to remission, which may contribute to increased cost of care.

**Aim:**

We explored perspectives regarding reasons for medication non-adherence among readmitted psychiatric patients.

**Setting:**

Weskoppies Psychiatric Hospital, Pretoria, South Africa.

**Methods:**

We adopted the social constructivism paradigm for this exploratory qualitative study. Purposive sampling was used to select 15 re-admitted patients, who were nonadherent to their medication. Data were collected through individual semi-structured interviews. The interviews were audio recorded and transcribed. The data were thematically analysed, using the principles of grounded theory.

**Results:**

Substance abuse, a lack of family support and poor health literacy were the most common reasons for non-adherence to medication. Other reasons included medication side effects, healthcare system drawbacks and a lack of finances to access healthcare. Some patients did not adhere to their medication because they believed that their mental illnesses were spiritual in origin.

**Conclusion:**

Multiple factors contributed to patients not adhering to their medication, ultimately resulting in their relapse and readmission. Clinicians should be cognisant of these factors when trying to prevent relapse and readmission.

**Contribution:**

Clinicians also ought to identify patients who are at risk of not adhering to medication. Targeted interventions should be established for tackling medication non-adherence.

## Introduction

Psychotropic medications are widely used and have shown to be effective for treating various psychiatric conditions.^1,2^ Non-adherence to psychotropic medications remains a significant problem globally.^3,4^ Factors contributing to medication non-adherence vary from patient to patient and may be intentional (missing and altering medication dosages) or unintentional (forgetting to take medication) or a mixture of both. The World Health Organization defines medication non-adherence as when people do not take their medication as recommended by their healthcare provider.^4^

Globally, studies show that only 50% of patients adhere to their chronic medication.^1,4^ In patients with severe mental illness, as many as 65% do not take their medication as recommended,^5^ with medication non-adherence rates differing according to psychiatric diagnoses.^3^ For example, patients with psychotic disorders are more likely to default treatment compared to those with mood disorders.^3^ Patients with high anxiety may also be more prone to poor adherence, because of fear of adverse medication effects.^6^ Medication non-adherence may exacerbate mental illness, increase the risk of suicide, lead to poor quality of life, result in additional comorbid medical conditions as well as increased relapse and re-hospitalisation rates.^5^ Furthermore, non-adherence is associated with longer time to remission, which leads to inflated inpatient care costs and excessive burden on caretakers.^7,8,9^

In high-income countries such as Europe and the United States, poor insight into mental illness and treatment, negative attitude towards medication, substance abuse, poor therapeutic alliance, unemployment, impaired social functioning, and self-stigma are some of the reasons for poor adherence to medication.^10,11,12^ Adherence to medication is reportedly lower in low-income countries.^1,4^ In studies performed in Ethiopia and Nigeria, additional factors such as poverty, inadequate social support, complex treatment regimen, medication side effects, poor insight, belief that illness is spiritual in origin, and being cured of mental illness have been linked to non-adherence.^13,14,15^

Many of the studies on medication non-adherence in South Africa have included patients in outpatient settings.^16,17,18^ In South Africa, anecdotal evidence suggests that medication non-adherence is common. Docrat et al.^19^ found that in South Africa, medication non-adherence contributes to 24% of readmission into inpatient care within 3 months of discharge. In South Africa, stable psychiatric patients are followed up at local community clinics and thus it is important that they adhere to their medication to reduce the risk of relapse and re-admission.^20^ In Mpumalanga, in the rural area of the Mmametlhake health district, Sharif et al.^16^ reported that people did not adhere to medication because of side effects. While in KwaZulu-Natal, uMgungundlovu district, Ramlucken and Sibiya^17^ found that patients in outpatient settings often forgot to attend their appointments, had work-related commitments, transport difficulties, or could not afford to attend visits.

Medication adherence has been assessed using various methods, which may be either direct or indirect. These methods include patient interviews, self-report, patient records, manual and automated pill counts, measurement of drug plasma levels, assessing patient clinical responses, and electronic devices (biosensors, short message service [SMS], and telemonitoring).^1,8,9^ Results from these methods have been ambiguous and no method has been shown to be superior.^1,4^ Using qualitative research findings may help policymakers tailor interventions to address the needs of target populations.^21,22^

A clear understanding of patients’ perspectives is crucial towards tackling medication non-adherence. Pubmed, Medline, and African Healthline search were carried out, but there seems to be paucity of qualitative research studies that explore perspectives on medication non-adherence in Southern Africa. This study explored the perspective on the reasons for medication non-adherence among patients who were discharged and subsequently re-admitted at Weskoppies Hospital.

## Research methods and design

### Study design

A qualitative research approach was chosen, as qualitative studies provide a deep understanding of complex human experiences and behaviours through the collection and analysis of non-numerical data.^23^ The study adopted social constructivism as the research paradigm. It is an interpretative framework that inductively generates meaning from subjective views or experiences.^23^ This exploratory qualitative study took place at Weskoppies Hospital, a tertiary psychiatric facility in Pretoria, South Africa. The hospital is in the Tshwane district of the Gauteng province. Weskoppies Hospital functions as both an inpatient and outpatient psychiatric facility, providing psychiatric services to children and adolescents, psychogeriatric, general adults, and forensic patients.

### Study population and sampling

Patients who were of interest to the study were purposively sampled. Fifteen patients were interviewed using individual semi-structured interviews. Patients between the ages of 18 and 60, who were able to give informed consent with a history of defaulting treatment and re-admission to Weskoppies Hospital were included in this study. Using routine hospital risk assessments tools, we excluded patients who were first contacts to psychiatric services, actively psychotic and aggressive or with neurocognitive disorders.

### Data collection

Prospective participants were identified from admission records, which were updated daily and from Medi-com archives (a health information system). Mental healthcare practitioners at the hospital were informed about the study and were requested to refer all eligible patients. Mental healthcare practitioners made of consultant psychiatrists and registrars in training, who conducted regular ward rounds for inpatients. Data were collected during the lockdown period because of the coronavirus disease 2019 (COVID-19) pandemic, and there were restrictions on close interpersonal contact. Infection control measures were observed during all interviews. Individual interviews took place in a well-ventilated room and social distancing was maintained. All people wore face masks and used hand sanitisers.

Initially, a brief session with individual patients was conducted, during which informed consent was obtained. During this session, the patients were given all the information and choices regarding the study. Individual interviews were conducted in either English, Setswana, or Sesotho. The principal investigator was fluent in all three languages. The interviewer was the principal investigator of the study. He worked at the same hospital where data collection was performed. The interview process ensured privacy and the maintenance of a clinically sound and therapeutic environment. Safety measures were in place in case of emergencies. Clinical data relevant to the study were extracted from patients’ clinical files.

Semi-structured interviews with guiding open-ended questions were conducted, and participants were allowed to express their perspectives regarding reasons for medication non-adherence. The interview guide included questions on demographics; the history of defaulting treatment; the reason that led to the current readmission; the description of previous admissions to psychiatric facilities, including the current and the reasons thereof; participants’ perspectives on any other reasons, other than medication non-adherence. Clarifying questions were asked to probe deeper depending on participant responses. Over time the interview guide was amended and became reflective of the varying responses. Most interviews were concluded within 45 min and provided rich data. Additional time was allowed where necessary. All interviews were audio recorded and field notes were taken, which added to the richness of the data. Those interviews conducted in Setswana and Sesotho were translated into English by the professional transcriber.

### Data analysis

Sociodemographic data were descriptively analysed and summarised as portions and percentages. The interviews were audio recorded and transcribed professionally by a transcriber who fluently spoke the mentioned languages. Transcripts and field notes were analysed using thematic analysis in which a coding system was used to identify recurrent themes that emerged from the data. Coding was performed manually to identify words that defined an idea from each interview. Trustworthiness was ensured through field notes and peer debriefing and member checking before data analysis.^23^ Similar codes derived from the segmented data were continuously redefined and followed by categorisation into emerging themes. Data saturation was reached after 15 interviews, when new information did not change the coding frame. The use of grounded theory principles ensured dependability of emerging themes. Abstract concepts were used to propose a theory as an explanation for a specific phenomenon (non-adherence to medication) and identify core theme.^24^

### Ethical considerations

The study was approved by the University of Pretoria, Faculty of Health Sciences Research Ethics Committee, reference no: 770/2020. Before participation in the study, signed informed consent was obtained from each participant. This study was explorative, and no harm was inflicted on the participants. The patient’s capacity to consent for participation was assessed clinically and with the use of a functional approach as recommended by Van Staden and Kruger.^25^ Participants were able to understand the study proposal, choose to partake or not, and communicated their choice.^25^ Only eligible participants took part in the study. To ensure confidentiality, participants remained unnamed and identified using a unique code. Data will be stored for 10 years according to the university policy.

## Results

In this study, 15 participants were interviewed, 10 men and 5 women. The participants’ ages ranged from 18 to 60 years old with 53.3% of people aged between 31 and 40 years. Over half of the participants had a secondary level education and 93.3% were unemployed. Most lived with their parents and were affiliated with the Christian religion. Among all participants, 40% had substance-related disorders. Most participants were readmitted three times or more (Table 1).

**TABLE 1:** Sociodemographic data of 15 participants who were interviewed regarding their reasons for medication non-adherence.

Variables	Frequency
**Gender**	
Male	10
Female	5
**Age (years)**	
18–30	3
31–40	8
41–50	2
51–60	2
**Level of education**	
Primary	4
Secondary	8
Tertiary	3
**Living conditions**	
With family	1
Parents	9
Alone	1
Other (Friends, Placements)	4
**Employment status**	
Unemployed	14
Employed	1
**Number of readmissions**	
One	3
Two	2
Three and more	10
**Religion**	
Christianity	14
**Other**	1
**Diagnosis**	
Schizophrenia spectrum	4
Bipolar and related	4
Depressive disorder	1
Substance use co-occurring with other psychiatric disorders	6

### Emerging themes

Participants reported various reasons that affected adherence to medication. Seven themes, each with subthemes emerged from the study and are illustrated in the next section with supporting verbatim quotations (Table 2).

**TABLE 2:** Themes and subthemes of the study.

Themes	Subthemes
1. Substance abuse	1.1. Cannabis use 1.2. Khat use
2. A lack of support from family	2.1. A lack of understanding of mental illness by family 2.2. Stigma
3. Poor health literacy	3.1. Patients’ limited understanding of mental illness 3.2. Doctors’ guidance not followed by patients
4. Medication-related side effects	4.1. Unfavourable medication side effects
5. Healthcare system drawbacks	5.1. Poor patient record keeping at clinics. 5.2. Inadequate communication regarding continuation of treatment. 5.3. Unavailability of medication at clinics
6. A lack of finances	6.1. Poverty of family support systems 6.2. Unemployment
7. Participants’ belief systems	7.1. Religious beliefs’ system 7.2. Traditional belief system

#### Theme 1: Substance abuse

The most common reasons for medication non-adherence among participants were cannabis, methcathinone, an amphetamine like stimulant and alcohol use. Participants mentioned substance use as a deterrent to medication adherence and they expressed such views as follows:

‘I think smoking cannabis makes me to come back and be readmitted. It does make me forget to take medication. It is like a dispatch it gives me hope and makes me forget my problems.’ (Participant 7, male, 36-years-old with schizophrenia, cannabis use disorder)

‘I also use Khat. Sometimes I go far for drinking. When I get there, we will be busy drinking and chatting away, then I wonder when I wake up if I did take my medication, or if I was scared to take it because it will affect the alcohol. If am not sure if I took it. I take the morning medication, instead of the night medication.’ (Participant 15, male, 31-years-old with schizoaffective disorder-bipolar subtype, stimulant and alcohol use disorder)

#### Theme 2: A lack of support from family

Participants mentioned having an unsupportive family as one of the most important reasons for medication non-adherence. A lack of understanding of mental illness and what seemed to be stigma contributed to a lack of support from families. Without family involvement, participants became despondent and discontinued medication as exemplified by the following views:

‘The most thing that makes me to relapse is sadness and not having support of my wellbeing and living with people who don’t support me that is why I relapse.’ (Participant 3, female, 39-years-old with major depressive disorder and substance use disorder)

‘I was taking my medication, but I won’t at times and wanted to talk to my parents about my condition to understand it better, they won’t be interested to talk to me. Sometimes they would be impatient when I talk to them. Someone in my condition does not understand the situation by himself, except to have people who surround him to be supportive.’ (Participant 5, male, 33-years-old with cannabis induced psychotic disorder and cannabis use disorder)

#### Theme 3: Poor health literacy

A lack of awareness of mental illness and importance of medication led to participants’ non-adherence. Failure to follow doctors’ guidance seemed to also contribute to non-adherence. The following quotes demonstrated participants’ views:

‘I didn’t quite understand what depression was all about. I just thought it was a state of mind and once you do the pills, and you feel better, you can just let it go at any point. So, I think my decision at that time was based on ignorance and I would have to live on meds for basically the rest of my life. So that freaked me out as well and I left the medication.’ (Participant 2, female, 26-years-old, bipolar I disorder)

‘Okay, I did not follow the doctor’s instructions. So, I ended up taking my medication on an empty stomach and sometimes not taking it all. I will forget to take it. So, in this instance, it took me to relapse.’ (Participant 4, female, 29-years-old, bipolar I disorder)

#### Theme 4: Medication-related side effects

Another frequently reported reason for discontinuing medication included unfavourable medication side effects. Participants mentioned various distressing side-effects including sedation, stiffness of body parts, and mental slowing. Participants expressed the following:

‘I think it’s mainly just skipping my pills every time I come here. I usually skip my pills, because I complained about being too sedated during the night and I couldn’t wake up to go to the bathroom, I end up wetting the bed. Also, my neck was stiff. Like I sprang my neck.’ (Participant 10, male, 31-years-old, schizophrenia, cannabis use disorder, stimulant use disorder)

‘Before all this I was not taking my medication, in fact, the more I take medications, the more I spend time on my bed. I would feel sleepy at work. I couldn’t be at my peak due to this medication because I would be sleepy, most of the time it was difficult for me waking up. I stopped taking medication for two years. So, I think I was more functional than when I’m drinking tablets. Because, when I take them, they restrict me, my memory will not be functioning effectively as I would like it to.’ (Participant 14, male, 41-years-old, bipolar I disorder)

#### Theme 5: Healthcare system drawbacks

Inadequate healthcare services were identified as a barrier to adherence. Reasons included missing patient files, medication stock outages, and poor communication regarding continuation of treatment. Participants expressed the following views:

‘It was like when I went to clinic, and they say that they didn’t see my files. They struggled to find it. So, I went back home. I didn’t have medication for two weeks.’ (Participant 5, male, 33-years-old, cannabis induced psychotic disorder and cannabis use disorder)

‘I was an outpatient and then they told me that, I do not need to take medication anymore.’ (Participant 9, male, 30-years-old with schizophrenia, cannabis use disorder)

‘When referred to the clinic there was no injection for three months. On the fourth month they told me that there is no medication neither injection then I started to get sick.’ (Participant 12, male, 51-years-old, schizophrenia)

#### Theme 6: A lack of finances

Poverty of family support systems and unemployment seemed to play a role in medication non-adherence. Participants mentioned not having money as an important reason for being unable to access healthcare services and expressed the following views:

‘I started to be sick. I did not have money to come to take medication and that made me to relapse.’ (Participant 6, female, 29-years-old, bipolar related disorder due to another medical condition)

‘Before I went to Mamelodi hospital my Epilim was finished, and my mother did not have money to give me to come to OPD [*Outpatient department*] at Weskoppies for three months exactly. She fell ill and couldn’t come with me.’ (Participant 11, male, 35-years-old, bipolar I disorder)

#### Theme 7: Participants’ belief systems

Although inconsistent across participants, religion and what may be described as traditional belief systems seemed to contribute to medication non-adherence. For instance, some participants believed that hearing voices is a form of a spiritual gift or a calling and expressed the following views:

‘My story is twofold. I have an aunt who doesn’t want me to be initiated. Like, it’s a cultural thing. There are things that I have to do on my own and she doesn’t support. You understand? I have a calling. So I get some instructions from my ancestors on what to eat and not to eat. So, the treatment was somehow in a way. So they asked me to stop.’ (Participant 1, male, 43-years-old, cannabis induced psychotic disorder)

‘I don’t believe that I’m ill. This is something normal that is happening to me. Hearing voices is normal. It depends on one’s school of thought. So, if you are a religious person, you will understand that these things happen due to a gift. It might be a spiritual gift. So, when you’re reasoning out with science, they will say it is chemical imbalance.’ (Participant 14, male, 41-years-old, bipolar I disorder)

## Discussion

This study qualitatively explored perspectives on reasons contributing to medication non-adherence among patients who were readmitted at a tertiary psychiatric hospital. The results showed that medication non-adherence was related to various factors, including comorbid substance use, a lack of family support, impaired insight, poor health literacy, financial constraints, and belief systems. Anecdotally, some patients who are re-admitted at Weskoppies Hospital have a history of defaulting their medication. These patients may take longer to respond to subsequent treatment, resulting in prolonged hospitalisation.^7^

Similar to this study, other studies have shown that patients with severe mental illness and comorbid substance use have a higher risk of non-adherence.^26^ About 50% of patients with comorbid substance use discontinue medication within the first few weeks of starting treatment.^27^ In the referenced study, it is unclear if substance use and non-adherence may contribute to each other or if the association is because of individuals’ personality traits.^28^ Participants in this study reported using cannabis, alcohol, and methcathinone for recreational purposes and as a coping mechanism. Our findings suggest that using these substances may contribute to a person forgetting to take medication and may lead to subsequent relapse and readmission. Our findings support previous studies illustrating that substance use negatively affects adherence to prescribed medications for patients with mental illness.^29,30^ In contrast, Okpataku and co-workers^31^ reported that substance use did not influence medication adherence in Nigeria; rather, medication adherence was influenced to a greater degree by unavailability of medication. This study found that substance abuse was experienced as a contributing factor to non-adherence.

Various studies have identified a lack of family support as another factor contributing to medication non-adherence.^13,14,32^ Participants in our study reported that their families were not there for moral and emotional support, which caused them to feel despondent and stop their medication. Some participants lived with their parents, illustrating that these patients struggled to live independently. A conducive family environment thus plays a significant role in patients’ well-being. Caregivers, however, experience a large burden and caregiver burnout, which may explain the waning care that occurs over time.^33^

Impaired insight has also been linked to medication non-adherence.^28^ Poor health literacy frequently occurs when patients with mental conditions are unable to appreciate the need for medication and the nature of their illness.^32^ Poor insight into mental illness and expected treatment outcomes may contribute to non-adherence.^34^ In this study, some participants did not understand the chronic nature of their mental illness, and they expected a cure rather than long-term treatment. Some seem to discontinue treatment after symptom relief. Self-stigmatisation seemed to deter patients from taking their medication as well. This underscores the need for healthy therapeutic alliance and regular patient psychoeducation.

Financial constraints were also identified as an important factor contributing to medication non-adherence. Some participants in this study stopped taking medication because they had no money to collect medication from healthcare facilities. The lack of adherence may be further exacerbated by a high rate of unemployment generally in our study setting,^35^ and with mentally ill people specifically.^36^ Financial incentives may improve adherence in psychiatric patients who are on chronic medication.^37^ In South Africa, disability grants facilitated by the South African Social Security Agency (SASSA) could enhance adherence.^38^ There are studies in agreement that social welfare and treatment adherence may be synergistic, and thus results in a greater cost efficiency.^39,40^

In addition, certain patient factors may affect medication adherence, including belief systems. In this study setting, some participants believed that their mental illness was spiritual in origin and could not be cured with western medication. Also, some participants mentioned having a cultural calling of being a healer or that their illness was a religious spiritual gift. Furthermore, a recent review suggested that the effect of spirituality/religiousness on mental illness was bidirectional and was associated with a better sense of self and coping with distress.^41^ The findings are similar to studies elsewhere. In Nigeria, in a study on medication non-adherence, in a tertiary psychiatric hospital, patients believed that their psychiatric illness had spiritual origins and could not be treated with orthodox medication.^42^ In clinical practice, inquiry into spirituality or religion is important and contributes to the provision of holistic and patient-centred care.

Medication side effects also led to the discontinuation of medication in this study. Participants mentioned neck stiffness, cognitive dulling with drowsiness and daytime fatigue that interfered with their productivity. Similar to this study, medication-related side effects have been well documented in other studies.^10,14,16,18,42^ Side effects from medication may affect patients’ quality of life and thus perpetuate a further barrier to adherence.^22^ Medication selection should consider patients’ target symptoms and side effect profile to promote adherence.^1^ Other medication-associated factors include stock outages, which resulted in participants not receiving their treatment when they visited local clinics.^43^ Missing records also seemed to contribute to delayed intervention, poor communication regarding follow ups, and duration of treatment. A similar study has reported flaws in the South African health system that were related to patients defaulting their mental health treatment while being admitted to hospital for other conditions.^18^ Interventions that ensure continuity of care such as adequate allocation of resources in primary care settings, skills training for healthcare providers working with the mentally ill, regular enquiry on medication patients are taking for comorbid illness, and efficient running of healthcare facilities may improve healthcare delivery in South African settings.^44^

### Recommendations

The following recommendations emerge from the study:

- Further studies on medication non-adherence in developing countries may contribute positively to healthy living and relapse prevention in persons with mental illness.
- Interventions should address substance misuse as a modifiable risk factor through routine screening and referral of patients to substance rehabilitation services during and beyond hospitalisation.
- Family supportive therapy and guidance may resuscitate caregivers’ support towards family members with mental illness.
- Healthcare providers should refer eligible patients for financial relief.

### Limitations

Our findings cannot be generalised to other settings as our findings are confined to one hospital and may not necessarily represent other hospitals in the region. This was a qualitative study, and the findings are thus subjective to the experiences of the participants.

## Conclusion

The study explored the reasons for medication non-adherence among patients readmitted to Weskoppies Psychiatric Hospital. The reasons for non-adherence were multifactorial and were related to socio-economic aspects such as substance abuse, a lack of support from family, and access to healthcare because of financial constraints. In addition, medication side effects and healthcare system drawbacks contributed to discontinuation of medication. Patients also defaulted on their medication because of poor health literacy and the belief systems that their mental illnesses had spiritual origins. The study highlights the importance of routinely assessing patients at risk, as well as the importance of establishing targeted interventions for tackling medication non-adherence.
